# Acculturation and depression increase trouble sleeping in Mexican immigrant adults

**DOI:** 10.1371/journal.pone.0311288

**Published:** 2024-10-18

**Authors:** Cameron K. Ormiston, Diana Lopez, Francisco A. Montiel Ishino, Timothy S. McNeel, Faustine Williams

**Affiliations:** 1 Division of Intramural Research, National Institute on Minority Health and Health Disparities, Bethesda, MD, United States of America; 2 Department of Medical Education, Icahn School of Medicine at Mount Sinai, New York, NY, United States of America; 3 Information Management Services, Inc., Calverton, MD, United States of America; University of Helsinki, FINLAND

## Abstract

Knowledge of Mexican immigrant sleep health is limited. We investigated the association between acculturation, depression, and having trouble sleeping among a nationally representative sample of Mexican immigrant adults. We used a logistic regression model on cross-sectional data from the 2005–2018 National Health and Nutrition Examination Survey on 2,670 non-U.S.-born Mexican adults aged ≥18 years old. Living in the U.S. for ≥10 years (Adjusted Odds Ratio (AOR) = 2.18; 95% Confidence Interval (CI) = 1.39–3.41), speaking majority English (AOR = 1.62; 95% CI = 1.00–2.64), and mild (AOR = 2.70; 95% CI = 1.82–4.02), moderate (AOR = 3.96; 95% CI = 2.53–6.19), and moderately severe/severe (AOR = 5.75; 95% CI = 3.08–10.75) depression levels were associated with having trouble sleeping. Non-U.S. citizenship status was associated with lower odds of having trouble sleeping (AOR = 0.62; 95% CI = 0.43–0.88). Greater acculturation and depression are associated with higher odds of having trouble sleeping. We provide new knowledge on how citizenship status may be linked to the sleep health of Mexican immigrant communities.

## Introduction

Insufficient sleep is considered a public health epidemic in the United States (U.S.) [1]. In fact, more than 50% of U.S. adults have difficulty sleeping, 30–40% experience insomnia symptoms annually, and approximately 20% meet diagnostic criteria for insomnia [2]. Insomnia symptoms include having trouble sleeping/falling asleep (difficulty transitioning from being awake to sleeping, restlessness, taking longer than 20–30 minutes to fall asleep), maintaining sleep, and having nonrestorative sleep [3, 4]. Additionally, the deleterious impacts of insomnia and sleep disruptions on health and disease risk have been well-documented in literature [5, 6]; insomnia is associated with excess caloric consumption, metabolic syndrome, impaired immune system, 24-hour sympathetic nervous system arousal, and abnormal hormone and metabolism regulation [6]. What’s more, insomnia elevates the risk of chronic disease, obesity, and mental health disorders [6]. For example, men with insomnia have double the risk of developing prostate cancer, while women with insomnia have nearly two times the risk for breast cancer [6]. The association between insomnia and depression is well-documented in a variety of study designs and populations [7–11]. For example, a global study found sleep problems were associated with almost 2.5x higher odds of brief depressive and 3.6x higher odds of depressive episode [12].

Not much is known about the sleep health of Mexican immigrants since most research has focused on the general Hispanic/Latino and non-Hispanic White populations [13, 14]. Evidence shows insomnia is undertreated in the Hispanic/Latino population and Hispanics/Latinos may bear a disproportionate burden of insomnia symptoms [15, 16]. Additionally, the prevalence of insomnia among Hispanics/Latinos may be higher than currently reported as they are less likely to report sleep complaints due to varying levels of sleep symptom knowledge [16, 17]. Hispanics/Latinos are also more likely to have high-risk sleep characteristics, such as excessive daytime sleepiness [14]. Moreover, it has been suggested that insomnia is a unique risk factor for cardiometabolic diseases within the Hispanic/Latino population [14, 16]. Additional research has shown Hispanic/Latino Americans with insomnia symptoms report significantly poorer mental health than White Americans [18]. Racial/ethnic inequities also exist across age groups in insomnia severity—Hispanic adults experience more insomnia severity with increasing age when compared to non-Hispanic White adults [19].

Insomnia and trouble sleeping has been found to be determined by a person’s psychosocial and environmental factors, meaning acculturation (the process of adopting or borrowing traits from a host culture, thereby modifying an individual’s or group’s culture; often defined as language(s) spoken at home, years lived in the U.S., citizenship status) may influence the risk of sleep troubles among diverse communities [20, 21]. In a cross-sectional study of low-income women (74% Hispanic, 8% non-Hispanic Black) in South Texas, language-based acculturation was associated with poor sleep quality and shorter sleep duration [22]. Among Hispanic/Latino adolescents and adults, increased acculturation has been associated with worse sleep outcomes [23–25]. Grandner et al. [17] analyzed the 2007–2008 National Health and Nutrition Examination Survey (NHANES) and found Mexican-born immigrants had a significantly lower risk of reporting difficulty falling asleep compared to U.S.-born respondents, a trend the authors believe is explained by acculturation. It should be noted, however, that the U.S.-born respondent category included non-Hispanic/Latino groups. A study comparing U.S.-born Mexicans and Mexican-born immigrants found U.S.-born Mexicans report more insomnia, which is likely due to higher rates of acculturation [26]. Other studies have also examined the association between acculturation, mental disorders, and insomnia [7, 24]. For example, Manber, Steidtmann (7) found increased depression severity and acculturation levels (defined by language use) were associated with higher insomnia risk in pregnant low-income Latinas. Similarly, it has been suggested that higher acculturation levels and depressive symptoms contribute to sleep disturbances in low-income women of Mexican descent [24]. Additionally, an analysis of Mexican American adults in the 2005–2018 NHANES reported higher acculturation and moderately severe/severe levels of depression were significantly associated with short sleep duration and long sleep duration [27].

There is currently a paucity of work investigating the association between acculturation, depression, and trouble sleeping (an insomnia symptom) within the Hispanic/Latino population, despite it being the largest immigrant population in the U.S. [16]. This study aimed to address this research gap by analyzing these associations using a nationally representative sample of Mexican immigrant adults. We hypothesized that (1) acculturation will be positively associated with trouble sleeping and (2) depression will exhibit a positive relationship with trouble sleeping.

## Methods

### Study sample and data source

Data from the 2005–2018 NHANES were obtained in August 2020 and analyzed from August 2020 to September 2020. The authors did not have access to information that could identify individual participants during or after data collection. NHANES combines interviews and physical examinations, and it is designed to produce estimates that are representative of the noninstitutionalized civilian U.S. population. Analyses were limited to respondents who reported Mexican origin or ancestry and being born outside the 50 US states or Washington, D.C. The sample was further limited to adults aged ≥18 years who received the physical examination (3,787). Subjects whose answers were provided by a proxy or who had missing values for any variables used in these analyses were excluded (n = 1,117), for a final sample size of 2,670. More information on the recruitment, design, and survey questions for the NHANES can be accessed on the Centers for Disease Control and Prevention—National Center for Health Statistics website [https://wwwn.cdc.gov/nchs/nhanes/default.aspx]. Institutional Review Board approval was not needed since secondary data was used and no human subjects were involved.

### Measures

#### Trouble sleeping outcome

Having trouble sleeping came from responses to the question “Have you ever told a doctor or other health professional that you have trouble sleeping?” Responses were categorized as yes or no.

### Independent variables

#### Acculturation

Length of time in the U.S. was dichotomized as <10 years or ≥10 years based on the participant’s response choice (<1 year, 1 to <5 years, 5 to <10 years, 10 to <15 years, 15 to <20 years, 20 to <30 years, 30 to <40 years, 40 to <50 years, and ≥50 years) to how long they have lived in the U.S. [27, 28]. Language(s) spoken at home came from responses to the question “What language(s) do you usually speak at home? Do you speak only Spanish, more Spanish than English, both equally, more English than Spanish, or only English?” Responses were categorized as majority Spanish (Only Spanish; More Spanish than English), English and Spanish equally, and majority English (More English than Spanish; Only English) [27].

#### Depression

Depression was assessed via the 9-item Patient Health Questionnaire (PHQ- 9) [29], which has been validated in a variety of populations, including the general US population, Mexican adults, Latinos, and Mexican immigrants [29–34]. Individuals were asked “Over the last 2 weeks, how often have you been bothered by the following problems:” (1) Have little interest in doing things, (2) Feeling down, depressed, or hopeless, (3) Trouble sleeping or sleeping too much, (4) Feeling tired or having little energy, (5) Poor appetite or overeating, (6) Feeling bad about yourself, (7) Trouble concentrating on things, (8) Moving or speaking slowly or too fast, (9) Thought you would be better off dead [29]. The possible response choices for each item included: Not at all, Several days, More than half the days, and Nearly every day. Depression severity was found by summing the 9 items, each of which are scored from 0–3, for a total score of 0–27. Depression severity levels were scored as either minimal (0–4), mild (5–9), moderate (10–14), or moderately severe (15–19) to severe (20–27) [29]. The standardized Cronbach’s coefficient α for the depression score was 0.82 (unstandardized, 0.82) overall (n = 2,670). For interviews in English (n = 830), the standardized Cronbach’s coefficient α was 0.79 (unstandardized, 0.78). For interviews in Spanish (n = 1,840), the standardized Cronbach’s coefficient α was 0.84 (unstandardized, 0.83).

#### Covariates and controls

Sociodemographic variables were age at screening (18–44, 45–54, 55–64, 65–74, ≥75 years old), sex (female, male), marital status (married/living with partner, widowed, divorced/separated, never married), family income (at or below poverty level, above poverty level), and U.S. citizenship status (yes/no). These covariates were selected based on prior epidemiological research on this topic and population [28, 35–37].

#### Statistical analysis

A logistic regression model was used to examine the association between acculturation and trouble sleeping and between depression and trouble sleeping. Analyses were weighted, and Taylor series linearization methods were used to account for the stratified, multistage, cluster sample design of NHANES. Analyses were conducted using SUDAAN 11.0.3 [38]. Results are reported as frequencies, weighted percentages, adjusted odds ratios (AOR), 95% confidence interval (CI) at the 2-tailed level, and a statistical significance level of p < 0.05.

## Results

### Descriptive characteristics of sample

Our sample included 2,670 Mexican immigrant (born outside the U.S.) adults, and their sociodemographic characteristics are in Table 1. A majority of the sample was 18–44 years old (64.4%), male (55.4%), married/living with a partner (74.9%), above the poverty level (63.7%), and not a U.S. citizen (75.3%). Approximately 11.6% of the overall sample reported having trouble sleeping. For acculturation variables, 73.8% had lived in the U.S. for ≥10 years and 82.8% spoke majority Spanish. Regarding depression, 78.4% reported minimal levels, 15.0% mild, 4.8% moderate, and 1.8% moderately severe/severe levels of depression. Statistically significant differences were found across all categorical variables.

**Table 1:** Characteristics of sample, overall and stratified by trouble sleeping, NHANES 2005–2018 (N = 2,670).

	Overall Sample	Stratified by Trouble Sleeping		
	N (%^1^)	Yes n (%^2^)	No n (%^2^)	p-value
**Total**	2670	345 (11.6)	2325 (88.4)	
**Age at Screening (years)**				<0.001
18–44	1383 (64.4)	79 (6.2)	1304 (93.8)	
45–54	496 (19.0)	87 (18.8)	409 (81.2)	
55–64	440 (9.9)	94 (21.9)	346 (78.1)	
65–74	269 (4.9)	59 (25.3)	210 (74.7)	
≥75	82 (1.8)	26 (35.0)	56 (65.0)	
**Sex**				<0.001
Female	1307 (44.6)	220 (16.3)	1087 (83.7)	
Male	1363 (55.4)	125 (7.8)	1238 (92.2)	
**Marital Status**				<0.001
Divorced/Separated	265 (8.9)	53 (21.7)	212 (78.3)	
Married/Living with partner	1938 (74.9)	228 (10.4)	1710 (89.6)	
Never married	321 (12.9)	26 (7.1)	295 (92.9)	
Widowed	146 (3.3)	38 (27.4)	108 (72.6)	
**Family Income compared to Poverty Level**				0.003
Above poverty level	1656 (63.7)	238 (13.3)	1418 (86.7)	
At or below poverty level	1014 (36.3)	107 (8.5)	907 (91.5)	
**U.S. Citizen**				<0.001
No	1914 (75.3)	184 (8.4)	1730 (91.6)	
Yes	756 (24.7)	161 (21.1)	595 (78.9)	
**Length of time in U.S.**				<0.001
Less than 10 years	658 (26.2)	34 (4.1)	624 (95.9)	
10 years or more	2012 (73.8)	311 (14.2)	1701 (85.8)	
**Language(s) spoken at home**				0.007
Majority Spanish	2245 (82.8)	272 (10.6)	1973 (89.4)	
English and Spanish equally	258 (10.1)	38 (13.7)	220 (86.3)	
Majority English	167 (7.0)	35 (20.6)	132 (79.4)	
**Depression Severity**				<0.001
Minimal	2044 (78.4)	182 (8.1)	1862 (91.9)	
Mild	413 (15.0)	91 (21.0)	322 (79.0)	
Moderate	148 (4.8)	49 (28.8)	99 (71.2)	
Moderately severe/severe	65 (1.8)	23 (38.0)	42 (62.0)	

*Note*. Weighted prevalence shown as percentage

^1^Weighted column percent

^2^Weighted row percent

### Logistic regression for having trouble sleeping

Respondents living in the U.S. for ≥10 years had significantly higher odds of reporting trouble sleeping compared to respondents living in the U.S. for <10 years (AOR = 2.18; 95% CI = 1.39–3.41) (Table 2). Speaking majority English was associated with significantly higher odds for having trouble sleeping compared to speaking majority Spanish (AOR = 1.62; 95% CI = 1.00–2.64).

**Table 2:** Associations of acculturation and depression with trouble sleeping.

	Trouble Sleeping	
	AOR	95% CI (Lower, Upper)
**Length of time in U.S.**		
Less than 10 years	ref	-
10 years or more	2.18***	(1.39–3.41)
**Language(s) spoken at home**		
Majority Spanish	ref	-
English and Spanish equally	1.07	(0.73–1.58)
Majority English	1.62*	(1.00–2.64)
**Depression Severity**		
Minimal	ref	-
Mild	2.70***	(1.82–4.02)
Moderate	3.96***	(2.53–6.19)
Moderately severe/severe	5.75***	(3.08–10.75)

The results shown here came from one logistic regression model, which also included the covariates adjusted for age at screening, sex, marital status, family income, and U.S. citizenship status

*p < 0.05

**p < 0.01

***p < 0.001; AOR = Adjusted odds ratio; CI = Confidence interval.

Compared to minimal levels of depression, mild (AOR = 2.70; 95% CI = 1.82–4.02), moderate (AOR = 3.96; 95% CI = 2.53–6.19), and moderately severe/severe (AOR = 5.75; 95% CI = 3.08–10.75) levels of depression were all associated with significantly higher odds of reporting having trouble sleeping.

## Discussion

Consistent with our hypothesis, we found acculturation and depression levels were positively associated with reports of having trouble sleeping in Mexican immigrant adults. This trend may reflect the healthy immigrant effect and the uptake of negative health behaviors that arise due to acculturation and subsequently impact sleep quality [20]. Our study indicates acculturation and depression may play a harmful role in the odds of experiencing insomnia symptoms for our study population, however these conclusions are not definitive.

Paralleling a recent cross-sectional analysis of the National Health Interview Survey, the present study found increasing English language use to be associated with trouble sleeping among our sample [39]. Another study using the Study of Women’s Health Across the Nation reported higher English language use to be associated with 9% higher odds of reporting sleep complaints in Hispanics/Latinas [40]. English language use may also be a risk factor for insomnia, with Manber et. al [7] reporting greater use of English to be strongly associated with clinically significant insomnia in pregnant low-income Latinas. The authors hypothesized higher insomnia risk may be due to acculturation-related loss of social ties and group identity, which may also explain our findings [7]. For example, earlier socialization in the U.S. and English language preference have also been associated with sleep disturbances among women of Mexican descent in an urban California community [24]. Moreover, previous studies have reported English language use to be strongly associated with other poor sleep outcomes in Hispanics/Latinos, such as short and long sleep duration [20, 26, 41, 42].

Depression has previously been associated with insomnia in U.S.-born Mexican Americans and Hispanics/Latinos [7, 24, 26]. In fact, higher levels of depression are associated with insomnia risk in pregnant low-income Latinas and increasing depression severity is positively associated with trouble falling asleep in Latina immigrant women [7, 43]. This is consistent with the present study; however, the present study demonstrates this association in a nationally representative sample of Mexican immigrant adults. Depression severity is also linked to daytime sleepiness, severe insomnia, and poor sleep quality among a variety of Hispanic/Latino populations, including Latino farmworkers, Latina women, and a national sample of Hispanics/Latinos [44]. Existing literature indicates (1) poor sleep can be shaped by a person’s environmental and psychosocial context, and (2) evidence has suggested an association between acculturative stress—a decline in mental health status while undergoing acculturation [45]—and depression in Hispanics/Latinos [46–51]. As such, a possible issue to discuss in relation to our findings is acculturative stress and how it may influence the link between depression and sleep. For instance, it has been suggested that emotional distress (anxiety and depression) may be an underlying pathway through which acculturative stress affects sleep for Mexican immigrant individuals [52]. However, it should be emphasized that with the limitations of our model, this cannot be said with certainty and is purely a postulated mechanism.

## Limitations

Despite its strengths (i.e., use of a nationally representative sample of Mexican immigrant adults and large number of survey years), our study does have limitations. First, our study is cross-sectional, meaning we cannot draw any conclusions in terms of causality between acculturation, depression, and trouble sleeping. Also, our sample was relatively young, which may have influenced our findings. To increase our sample size, we used pooled data from NHANES 2005–2018, however a limitation of pooling data is that social contexts across survey years may be inconsistent and thus affect survey responses. Additionally, data on sexual orientation and gender identity were not included in our model, which would be vitally important to investigate for future research given known differences and inequities in health for sexual and gender minoritized populations [53]. Also, since there were no objective measures of having trouble sleeping available in the NHANES dataset, our data are based on self-reported measures, making them susceptible to recall and social desirability bias [54].

An additional limitation is our trouble sleeping variable was defined by a respondent telling a doctor or other health professional they have had trouble sleeping. Our results therefore may not capture Mexican immigrants who have trouble sleeping and are not connected with the healthcare system. Also, while the PHQ-9 has been used with Mexican, Mexican immigrant, and Mexican American populations, there may be variation in scoring cut points. The standard PHQ-9 scoring cutoffs were used for this study given our study population is comprised of individuals of varying migration stages. Future research should explore the variance in cutoff scores among different Mexican immigrant groups. Also, we were limited by the variables available in the NHANES for measuring acculturation. As a result, we were not able to fully capture the multi-dimensionality of acculturation, including diet, values, beliefs, relationships, and other cultural and psychosocial stress factors. Finally, acculturative stress as a discrete variable or measure was not available. Further longitudinal research is warranted to adequately explore this mechanism and determine causality [52].

## Future directions

Looking toward the future, depression is often comorbid with other physical and mental health conditions [55, 56]—which may play a role in the sleep health of Mexican immigrants and warrants further investigation. The use of imaging modalities in assessing sleep health is also a potential area of research as they may be more reliable than self-reported measures of sleep health. Importantly, research should investigate how acculturative stress could have influenced our findings given an analysis of the Hispanic Community Health Study/Study of Latinos (HCHS/SOL) Sueño and Sociocultural Ancillary studies by Alcántara, Patel (52) found acculturative and psychosocial stress are associated with insomnia symptoms (Relative Risk Ratio = 1.07; 95% CI = 1.04–1.11). A follow-up study from Alcántara, Gallo (1) using HCHS/SOL illustrated comparable results, showing each one standard deviation increase in acculturative stress was associated with a 0.75-point increase on the Insomnia Severity Index. The authors posited that acculturative stress leads to more insomnia symptoms by triggering emotional problems, worry, rumination, and vigilance that affects one’s ability to fall asleep [1].

## Practical and clinical implications

Our study findings highlight the importance of addressing acculturation and depression among Mexican immigrants. We also underscore the importance of health practitioners to screen for mental and sleep health issues among Mexican immigrants. Additionally, consideration of each patient’s sociocultural context must be given when tailoring health plans. Thus, clinical training centered on delivering culturally humble care is essential. Importantly, improving sleep health in the Mexican immigrant population can have far reaching positive health effects and work towards ameliorating health disparities [57].

## Conclusions

While prior studies have analyzed the link between acculturation and sleep health in Hispanics/Latinos, they typically generalize the Hispanic/Latino population or focus on other sleep variables, such as sleep duration. To our knowledge, no study has examined the association between length of stay in the U.S., citizenship status, depression, and having trouble sleeping among a nationally representative sample of Mexican immigrant adults. Ultimately, our work contributes to existing literature by highlighting the link between acculturation and depression and the sleep health of the Mexican immigrant population.
